# Examining the Mediating Effect of Customer Experience on the Emotions–Behavioral Intentions Relationship: Evidence from the Passenger Transport Sector

**DOI:** 10.3390/bs12110419

**Published:** 2022-10-29

**Authors:** Anastasia Gerou

**Affiliations:** Department of Maritime Studies, University of Piraeus, 18534 Piraeus, Greece; anais-gerou@hotmail.com

**Keywords:** customer experience, behavioral intentions, emotions, services marketing, transportation marketing, customer journey, coastal line shipping, travel and tourism, passenger transportation

## Abstract

The aim of this research is to examine the influence of customer experience on the customers’ emotions–customers’ behavioral intentions relationship. Moreover, this study seeks to obtain data that will help to address this research gap in the passenger transportation industry. A large-scale survey on the coastal line shipping industry was conducted. The random sampling methodology was employed, and the target respondents were ferry passengers. The anonymized questionnaires were completed by 840 passengers. The data were analyzed through exploratory and confirmatory factor analyses and regression analysis. The findings verified application of the adopted (customer experience, emotions) and adapted (behavioral intentions) measurement instruments in the examined customer journey in the passenger shipping sector and indicated the mediating role of customer experience in the relationship between customers’ emotions and customers’ behavioral intentions. A distinguishing feature of this work is that it measures and assesses emotions during the customer journey and not afterward based on recall journeys. Additionally, although academics have carried out extensive research on the emotions–behavioral intentions relationship, very little is known about the role of customer experience in this relationship. Thus, what is not yet clear is the impact of customer experience on the emotions–behavioral intentions relationship. Additionally, the measurement instruments used to test this research hypothesis were empirically tested for the first time in the passenger shipping service environment.

## 1. Introduction

The major issue of this paper is to determine the mediating role of customer experience on the customers’ emotions–customers’ behavioral intentions relationship in passenger transportation services. In recent years, there has been increasing interest in the study of customer experience [1], which is defined as a person’s subjective response to or interpretation of a company’s offerings while engaging in interactions with other people and the environment during the customer journey [2]. According to academics, marketing science studies, particularly customer management studies, have been reluctant to catch --up with the advances in marketing literature regarding customer experience [1]. Instead of concentrating on customer experience, customer management studies have mostly focused on customer value creation for businesses [3]. The growing emphasis on customer experience studies has developed from the complexity of customer-to-customer and customer-to-business interactions, which have emerged during the social media and multichannel modern era [4]. In 2016, the Marketing Science Institute emphasized the importance of customer experience, describing it as one of the most crucial research areas [1]. Although extensive research has been carried out on customer experience, and many have examined its role regarding customers’ behavioral intentions, no single study has surveyed the mediating role of customer experience in the customers’ emotions and behavioral intentions relationship.

The context of behavioral intentions in services describes customers’ willingness to recommend a service or brand, repurchase and purchase more of this service [5] and revisit the place where the service is provided. Behavioral intentions are an increasingly important area in service management and marketing since they are highly related to customer satisfaction [6,7] and constitute a component of customer loyalty [8,9]. Despite the importance of understanding customers’ behavioral intentions, there remains a paucity of evidence on how other factors, such as customer experience or customers’ emotions, affect customers’ behavioral intentions. A search of the literature reveals that no previous study has empirically investigated the relationship among those constructs in passenger transportation services.

There is a growing body of literature that recognizes the importance of emotions for customers’ behavioral intentions (e.g., Ref. [10]). Emotions are nuanced reactions to events [11]. At the intrapersonal level, emotions enable people to respond to significant events by influencing their cognition and attitude [12]. Interpersonally, emotions expressed by individuals contribute to social coordination by generating emotional, inferential and behavioral reactions from others [13]. Therefore, emotions have a major effect on our individual and societal life via these intrapersonal and interpersonal processes [14]. Although many researchers have recognized the critical role played by emotions on customers’ behavioral intentions, very few studies in the examined literature have measured customers’ emotions in the service environment during the customer journey and not afterwards based on recalled memories (e.g., Ref. [15]).

In light of the foregoing, this research aims to provide answers to the following questions: how can customers’ experience and the emotions and behavioral intentions of the customers be measured in passenger transportation services? Does customer experience mediate the customers’ emotions–customers’ behavioral intentions relationship?

Customer engagement is the overarching theory on which this study is based, and it emerged as the foundational ideas of marketing and consumer behavior evolved [16]. Customer engagement is defined as the degree to which a customer contacts and initiates interaction with a company, whether attitudinally or behaviorally [1].

The work described in this study is driven by the heightened awareness of academics that far too little attention has been paid to the role of customer experience in the relationship between emotions and behavioral intentions [17] as no single study exists that has examined this relationship so far. Additionally, relevant studies in the context of passenger transportation services are lacking.

The paper is divided into six sections. Section 2 provides a brief overview of the literature review and hypotheses formation. Section 3 analyzes the research methodology, design, measurement instruments and data. Section 4 scrutinizes the results of this study. In Section 5, the overall conclusions, theoretical and practical implications and avenues for future research are presented.

## 2. Literature Review and Hypotheses Formation

### 2.1. Customer Experience

#### 2.1.1. Definitions

Customer experience is defined as a person’s subjective response to or interpretation of a company’s offerings while engaging in interactions with other people and the environment during the customer journey [2]. It can be divided into six different types of responses: cognitive, emotional, behavioral, sensory, social and spiritual [18]. There are several definitions of customer experience in the literature [19] since customer experience is a multidimensional construct [20]. The distinction between customer experience and other consumer-related dimensions has been debated for many years among academic researchers. Through the years, researchers have tried to create definitions in which customer experience is differentiated from other similar constructs [21], such as service quality [22], customer satisfaction and customer loyalty [23], relationship management [24] and customer engagement [25]. In the examined bibliography, the term ‘customer experience’ is also referred to as ‘customer service experience’ [26].

#### 2.1.2. Historical Evolution

The variations among customer experience definitions are derived from the different scopes in which researchers have examined customer experience over the years. The origins of customer experience may be traced back to the 1960s, when the first key ideas of consumer behavior were established and articulated [27]. Afterwards, according to thorough analysis of Lemon and Verhoef (2016) [1], there have been significant advancements in and contributions to customer experience research. More extensively, in the decade of the 1960s, researchers focused on the process models of customer buying behavior, which were important for understanding how customers interacted with and made decisions about products and services [28]. In the 1970s, customer satisfaction and loyalty were determined by examining and evaluating the views and attitudes of customers about a product or service [29]. Furthermore, in the 1980s, academics tried to identify the specific context and features of a customer experience that were essential for service excellence [30]. In 1990s literature, relationship marketing studies contributed to the customer experience theory as the studies expanded the range of customer reactions that were taken into account in a customer experience [31]. In the 2000s, customer relationship management theory thrived, with the researchers using linkage models to determine how certain elements of a customer experience interacted with one another and how those elements affect the business results. In this decade, researchers have also concentrated on the transdisciplinary and organizational problems inherent in building and managing excellent customer experiences by conducting customer-centric studies [3,32]. From the beginning of the 2010s up to now, customer engagement theory has flourished in the literature, with researchers acknowledging the customer’s contribution to the experience [33,34]. 

#### 2.1.3. Measurement Instruments

Different authors have measured customer experience in a variety of ways. However, the most commonly applied measurement instruments of customer experience are the experience economy model of Pine and Gilmore, 1999 [35] and the model of the five dimensions of Schmitt, 1999 [36]. Both have been used in numerous articles that tried to measure customers’ experiences in accommodations, activities, destinations and restaurants. According to Kim et al. (2022) [20], Pine and Gilmore’s (1999) measurement instrument of customer experience is the most used scale in the scientific literature with regard to customer experience measurement in the tourism sector. Therefore, since this study examines customer experience in the ferry context, which is a combination of the transport and tourism sectors, the experience economy model of Pine and Gilmore (1999) is determined as the most appropriate, reliable and valid measurement instrument. Empirical studies using this measurement instrument in the bibliography of the examined service environment are lacking. In order to assess the mediating role of customer experience on the emotions–behavioral intentions relationship, we first have to ensure customer experience scale applicability in the ferry context. Thus, we posit that:

**[RH1].** **Research** **Hypothesis** **1.** *The onboard customer experience can be measured through a four-dimensional scale consisting of entertainment, education, escapism and aesthetic factors.*

However, to test the mediation effect of customer experience, we also have to verify the applicability of the emotion and behavioral intention scales on the examined service. 

### 2.2. Emotions

Through the years, many researchers have tried to define what emotions are. While the definitions are varied, there is little consistency among them, and many are not sufficiently specific [37] in order to offer a clear understanding of what an emotion genuinely consists of. Due to the absence of a definite description, several researchers have attempted to increase the understanding of emotions by elaborating on their elements. 

Clore et al. (1987) [38] provided one of the most precise definitions and one that looks to be gaining popularity among scholars. According to their definition, an emotion is ‘a valence affective reaction to perceptions of situations’. They removed from the definition of emotions adjectives referring to non-valenced cognitions (such as interest and surprise), physiological states (such as fatigued and drooping) and subjective appraisals of persons (such as self-confident or abandoned). Similar to Clore et al. (1987) [38], Cohen and Areni (1991) [39] also used the affective notion of emotions in their definition and described them as ‘affective states characterized by episodes of intense feelings associated with a specific referent and instigate specific response behaviors’, whereas Niedenthal et al. (2017) [11], based on the valenced tone of the Clore et al. (1987) [38] definition, described emotions as ‘valenced responses to situations that entail synchronized patterns of appraisals, experiences, physiological changes, expressions, and/or behavioral tendencies’. Abdelkader and Bouslama (2014) [40] stated that ‘emotions have some degree of intensity-positive or negative-associated to a stimulus that disrupts a stable state of the individual’. Through their definitions, these researchers have approached emotions from different perspectives and highlighted the positive and negative intensity of emotions.

In the literature of emotions, there are two basic theoretical perspectives: dimensional and categorical. The dimensional approach is valence-based, whereas the categorical approach refers to emotion specificity [41]. In the marketing and management literature, both the dimensional and categorical theories are preferred for measuring emotions, although the categorical theory is the most widely used conceptualization in the tourism bibliography. Despite this, there has been little quantitative analysis of customers’ emotions in the context of transportation services. Therefore, in this study, the categorical measurement instrument of emotions is adopted, and the following hypothesis is formulated:

**[RH2].** **Research** **Hypothesis** **2.** *The customers’ emotions in passenger transportation services can be measured through a two-dimensional scale consisting of positive emotion and negative emotion factors.*

### 2.3. Behavioral Intentions

Customers’ behavioral intentions can be classified into two categories: positive and negative intentions. The customers’ positive behavioral intentions in the service sector often include repurchase (or purchasing more), recommendations/word of mouth and revisitation, while their negative behavioral intentions include complaints, dissatisfaction with the firm and spending less time in the business [5].

Through the years, many researchers have tried to define what behavioral intentions are, and various definitions of behavioral intentions have been found (e.g., Refs. [42,43]). Although differences of opinion still exist, there appear to be two theoretical approaches among scholars regarding the definitions of behavioral intentions. The first category of definitions describes behavioral intentions as an outcome of the potential formation of customer behaviors (e.g., Refs. [42,44,45]), while the second category of definitions describes the term by elaborating on its elements (e.g., Refs. [5,43,46]). Those elements are repurchasing [46], repatronizing [43] and/or purchasing more [5] and the probability and willingness of a customer to say positive things (positive word of mouth) and recommend [5,46] the product, service and/or brand to other people. In line with the theoretical and empirical evidence in behavioral intentions studies, and since there is no tested measurement instrument of behavioral intentions that includes all the aforementioned behavioral intention elements, an adaptation of the Brown et al. (2005) [47], Li and Petrick’s (2008) [48] and the Rather et al. (2022) [49] behavioral intention scales is made. Thus, we submit the following hypothesis:

**[RH3].** **Research** **Hypothesis** **3.** *Behavioral intentions can be measured through a one-dimensional scale reflecting the customers’ word of mouth, repurchase and revisiting intentions.*

### 2.4. The Role of Customer Experience in the Emotions and Behavioral Intentions Relationship

The impact of customers’ emotions on their behavioral intentions is a major area of interest within the field of service management and marketing. Through the years, researchers have analyzed this relationship, and many studies have verified the strong impact of emotions on customers’ behavioral intentions. More extensively, studies in the 1970s and 1980s began to show the importance of emotions for behavioral intentions by assessing how positive emotions were associated with positive outcomes and behaviors (satisfaction and repurchase behavior) and how negative emotions were associated with negative behaviors (dissatisfaction and avoidance behavior) [50,51]. In the 1990s, researchers proved that emotions impacted service performance outcomes [52] and were critical factors of post-consumption behaviors [53]. In 2004, Zeelenberg and Pieters [54] stated that, when academics examined variables such as behavioral intentions, emotions were deemed significant, and, in 2005, Bigne et al. [55] verified this significance in the tourism industry by proving that emotions affected tourists’ behavioral intentions. Researchers also established that, in the post-consumption stage, emotions affected satisfaction [56] and loyalty [57], whereas, in the pre-consumption stage, emotions affected the customer’s decision to purchase [58], trust and commitment [59]. Thus, emotions must be taken into account when assessing indicators such as satisfaction [60] and behavioral intentions [61]. De Rojas and Camarero (2008) [62] and Del Bosque and San Martin (2008) [63] confirmed the impact of emotions on tourists’ satisfaction, and Hosany and Prayag (2013) [64] proved that positive emotions resulted in more favorable behavioral intentions (loyalty and desire to spend more) and satisfaction for tourists. Additionally, customers’ positive and negative emotional states had an effect on their behaviors [65]. According to Fast et al. (2012) [66], excessiveness in customers’ positive emotions might result in overconfidence, which is associated with negative outcomes. More recently, researchers have stated that emotions are critical in determining customers’ reactions [5,41] since they have a strong impact on their behavioral intentions [10].

Apart from the relationship between emotions and behavioral intentions, the relationship between emotions and customer experience is also a major area of interest among the service management and marketing literature. Academics have examined the impact of emotions on customer experience (e.g., Refs. [10,67,68,69]), and through many robust studies, it has been verified that customer experience is heavily influenced by emotions [41].

Besides the relationships between emotions and customer experience and emotions and behavioral intentions, researchers have been particularly interested in the relationship between customer experience and behavioral intentions. Academics began to examine this relationship in the 1990s and ascertained that customers gained more knowledge about products, services and companies through their experiences and their perceptions of these experiences, which allowed them to make comparisons with the companies’ competitors and, therefore, avoid unanticipated consequences [70,71]. In the last decade, studies proved that positive experiences may foster an emotional connection between businesses and customers, resulting in favorable customer behavioral reactions [72,73]. The customer experience influence on consumers’ behavioral intentions was demonstrated to be strengthened further when the customer experience was improved concurrently [74,75], while frequent negative customer experiences were shown to be connected with termination of the relationship between the customers and the company [9].

In addition to investigating the complete behavioral intention construct, researchers have also focused on its dimensions and analyzed the effects of customer experience on customers’ loyalty, word of mouth and recommendations and intentions to purchase and purchase more. More extensively, customer experiences have been found to have an effect on a customer’s intention to remain loyal [9]. Brakus et al. (2009) [76] were the first to reveal the existence of a strong relationship between customer experience and loyalty intentions. Additionally, regarding the relationship between customer experience and word of mouth/recommendations, many researchers have examined this connection (e.g., Ref. [77]) and verified that customer experience has a significant impact on customers’ word of mouth behavior [9] and recommendations [76]. Last, regarding the relationship between customer experience and customers’ purchase intentions, many academics have assessed their interrelation (e.g., Ref. [78]) and proved that customer experience has a significant impact on customers’ repurchase intentions [79] and customers’ purchase-more intentions [9].

Therefore, from the previous discussion, it is evident that a considerable amount of literature has been published on the impact of customer experience on behavioral intentions and the impact of emotions on customer experience and behavioral intentions. However, far too little attention has been paid to the role of customer experience in the relationship between emotions and behavioral intentions, and no single study exists that has examined this relationship so far. Recently, Chen and Yang (2021) [17] highlighted the need for examining the role of customers’ experience in relation to customers’ emotions and intentions. Thus, our paper aims to contribute to this growing area of research by exploring this impact. Following the above discussions, the fourth research hypothesis is:

**[RH4].** **Research** **Hypothesis** **4.** *Customer experience mediates the relationship between customers’ emotions and behavioral intentions.*

## 3. Data and Methods

### 3.1. Sample and Data Collection

A large-scale survey on the coastal line shipping industry was conducted. The random sampling methodology was employed, and a structured questionnaire was administered to 840 respondents, of which 455 were male (54.2%), 379 were female (45.1%) and 6 were non-binary (0.7%). Their ages ranged from 16 to 83 years, with a mean of 49.57 years. The respondents represented a wide range of nationalities: Greek (40.2%), German (21.1%), Italian (7.9%), Swiss (6.4%), French (6.1%), Austrian (4.4%), British (2.9%), Belgian (2.5%), Netherlands (2.5%), Albanian (2.1%), Croatian (1.1%), Russian (0.7%), Spanish (0.7%), Bulgarian (0.4%), Canadian (0.4%), Danish (0.4%) and Irish (0.4%). The respondents were tourists embarked on a passenger RoRo vessel, traveling from Greece to Italy and from Italy to Greece (total selection from 4 itineraries; each itinerary lasts 23 h). The data collection occurred in August 2021, and the survey consists of three parts. Part 1 aims to measure passengers’ emotions, part 2 aims to assess the customer experience and the behavioral intentions of the respondents and part 3 evaluates the personal characteristics of the respondents. The questionnaires had been prepared in three languages (English, Greek and Italian) based on the assumption that the majority of the passengers are able to communicate in those languages.

### 3.2. Measurement Instruments

To measure the customer experience in the service environment, Pine and Gilmore’s (1998) [35] experience economy model was used. The measurement instrument consisted of 12 items, which were organized in four dimensions: educational, escapist, aesthetic and entertainment experiences. Emotions were assessed using seven items organized into two axes: a. positive emotions and b. negative emotions [41]. The first three items reflected positive emotions (contentment, happiness and love), while the last four items were used to measure negative emotions (anger, fear, sadness and shame). Last, to measure the customers’ behavioral intentions, an adaptation of the measurement instruments developed by Brown et al. (2005) [47], Li and Petrick (2008) [48] and Rather et al. (2022) [49] was made. The behavioral intention construct included one factor and seven items. All the measurement items among the examined scales were measured on a seven-point Likert-type scale ranging from ‘strongly disagree’ to ‘strongly agree’. The questionnaire is included in Appendix A.

### 3.3. Methodology

Once it had been determined that all the scales had satisfactory homogeneity (using Cronbach’s alpha), “exploratory factor analysis” (EFA) was applied to find the dimensionality of the scale of customers’ behavioral intentions. Furthermore, a “confirmatory factor analysis” (CFA) was conducted for the customers’ behavioral intention measurement instrument to confirm the number of the construct’s dimensions. Regarding the other constructs examined in this study (emotions and customer experience), they have been extensively used and tested in the service sector; therefore, an EFA was not necessary. However, to confirm the existence of the number of dimensions suggested in the scientific literature and their applicability in the coastal line shipping industry (test of Research Hypothesis 1, Research Hypothesis 2 and Research Hypothesis 3), CFA was utilized with the maximum likelihood estimation. The mediating effect of customer experience on the relationship between customers’ emotions and customers’ behavioral intentions (test of Research Hypothesis 4) was tested through regression analysis following the procedure proposed by Baron and Kenny (1986) [80].

## 4. Results 

### 4.1. Testing the Examined Scales (Research Hypotheses 1, 2 and 3) 

An exploratory factor analysis using principal axis factoring was conducted to reveal the dimensionality of the scale of the customers’ behavioral intentions. The rationale for choosing EFA is that the scale is exploratory and comprises quantitative variables. As discussed in the literature review chapter, there is no comprehensive theoretical framework with regard to the different underlying items. This analysis generated a number of constructs, which, while not being correlated, explained the greatest percentage of the total variance [81]. 

First and foremost, it was considered whether it was sensible to conduct factor analysis on the correlation matrix. This question was answered by checking the results from Bartlett’s test as well as the Kaiser–Meyer–Olkin Measure of Sampling Adequacy (KMO-MSA) [81]. In this EFA (see Table 1), Bartlett’s test was significant (*p*-value < 0.05), indicating that it was appropriate to factor-analyze the matrix (as significance indicates that the sample correlation matrix is significantly different from an identity matrix). The KMO-MSA (KMO-SMA = 0.832, KMO-SMA > 0.5) indicated that the matrix was acceptable for factoring. According to Kaiser and Rice’s (1974) [82] terminology, the factorability of this matrix was considered ‘meritorious’ [KMO-MSA: >0.5 (miserable), >0.6 (mediocre), >0.7 (middling), >0.8 (meritorious) and >0.9 (marvelous)]. Last, there was no multicollinearity since the determinant of the correlation matrix (determinant = 0.001) was greater than 0.00001 [83]. Considering these results, this construct was appropriate to be factor-analyzed. 

The EFA results showed that only one factor characterized the behavioral intention construct (see Table 2). 

CFA was employed using maximum likelihood estimation in order to confirm the structures of the customer experience, emotion and behavioral intention constructs. The customer experience scale consisted of four factors, namely entertainment, education, escapism and aesthetic, while the emotion scale consisted of two factors—positive emotions and negative emotions. Last, the behavioral intention scale consisted of one factor, namely behavioral intentions. In all the examined constructs, Cronbach’s alpha value was above 0.7 (customer experience: 0.925, emotions: 0.827 and behavioral intentions: 0.908), which indicated a very satisfactory level of construct reliability, according to Hair et al. (2006) [81]. All the standardized regression weights were statistically significant, which confirmed the convergent validity of the measurement models. The absolute and incremental fit indices were highly satisfactory, thus signifying a very good fit. All the chi-square/df ratios fell within the acceptable range of 0–5 [84]. The root mean square error of approximation (RMSEA) was below 0.07 [85], while the goodness of fit statistic (GFI), adjusted goodness of fit statistic (AGFI), comparative fit index (CFI), normed fit index (NFI) and non-formed fit index (NNFI)—also known as the Tucker–Lewis index (TLI)—were very close to 1. The GFI and AGFI values were greater than 0.9 and the NNFI (TLI), NFI and CFI values were greater than 0.95 [86], which indicated a very good model fit. As a result, the absolute and incremental fit indices of the examined constructs (see Table 3) were highly satisfactory, thus confirming good measurement properties of all the examined instruments. The findings above indicated that:

- Research Hypothesis 1 has been supported, with the onboard customer experience scale composed of four distinct dimensions: entertainment (three items), escapism (three items), education (three items) and aesthetic (three items).
- Research Hypothesis 2 has been supported, and the emotion construct in passenger transportation services is composed of two distinct dimensions. The first one (positive emotions) expresses the positive emotions of the customers (whether they feel contentment, happiness or love), and the second one (negative emotions) expresses the negative emotions of the customers (whether they feel angry, sad, fearful or ashamed).
- Research Hypothesis 3 has been supported, and the customers’ behavioral intentions can be measured through one distinct dimension composed of seven items that reflect their word of mouth, repurchase and revisiting intentions.

### 4.2. Testing the Mediating Effect (Research Hypothesis 4)

**Figure 1:**
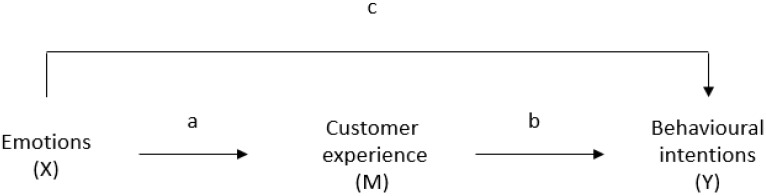
The examined mediating effect depiction.

The mediating effect of the customer experience can be expressed/depicted in the following way (Figure 1): paths a and b are the direct effects (a. emotions→customer experience and b. customer experience→behavioral intentions), whereas the mediation effect, in which X leads to Y through M (path c), is the indirect effect. The indirect effect in the examined hypothesis expresses the portion of the relationship between emotions and behavioral intentions that is mediated by the customer experience. The summated scale of behavioral intentions is utilized as the dependent variable. The summated scale of the four factors of the customer experience acts as the mediating variable, and the summated scale of the two factors of emotions is the independent variable.

The mediating role of effective implementation of customer experience in the connection between customers’ emotions and behavioral intentions was examined following the four-step process proposed by Baron and Kenny (1986) [80]. According to them, in order to test for mediation, four separate regression analyses must be performed:

- Step 1. A simple regression analysis with X (emotions) predicting Y (behavioral intentions) to test for path c [Behavioral Intentions = bo + b1 × Emotions + e].
- Step 2. A simple regression analysis with X (emotions) predicting M (customer experience) to test for path a [Customer Experience = bo + b1 × Emotions + e].
- Step 3. A simple regression analysis with M (customer experience) predicting Y (behavioral intentions) to test the significance of path b [Behavioral Intentions = bo + b1 × Customer Experience + e].
- Step 4. A multiple regression analysis with X and M predicting Y [Behavioral Intentions = bo + b1 × Emotions + b2 × Customer Experience + e].

The purpose of Steps 1 to 3 was to establish the zero-order relationships among the existing variables. In order for mediation to be possible, all the simple regressions had to be statistically significant. Moreover, in Step 4, some form of mediation would be supported if the effect of M in path b remained significant after controlling for X. If X was no longer significant when M was controlled, the finding would support full mediation. If X was still significant (i.e., both X and M significantly predicted Y), the finding would support partial mediation [80].

The correlation coefficients among the constructs of interest are shown in Table 4, while the results of the above-described regression equations are presented in Table 5, Table 6, Table 7 and Table 8.

The results of the analyses above revealed that there were statistically significant correlations between the examined constructs, so there was justification to proceed with the testing for the mediation analysis. All the simple regressions (Steps 1 to 3) were statistically significant (see Table 5, Table 6 and Table 7), and the multiple regression (Table 8) showed that both X and M significantly predicted Y. The finding supported mediation (partial). More specifically, all four conditions of Baron and Kenny (1986) [80] held true:

- In the first regression equation (Table 5), the customers’ behavioral intentions were affected by their emotions (Adj. R^2^ = 0.195) [Behavioral Intentions = 5.516 + 0.649 × Emotions + e].
- In the second regression equation (Table 6), the customers’ emotions had an impact on their customer experience (Adj. R^2^ = 0.143) [Customer Experience = 13.790 + 0.787 × Emotions + e].
- In the third regression equation (Table 7), the customers’ experience had an impact on their behavioral intentions (Adj. R^2^ = 0.386) [Behavioral Intentions = 10.706 + 0.440 × Customer Experience + e].
- In the multiple regression equation (Table 8), the customers’ behavioral intentions were affected by the customers’ emotions and customer experience (Adj. R^2^ = 0.435) [Behavioral Intentions = 0.340 + 0.353 × Emotions + 0.375 × Customer Experience + e].

**Scheme 1:**
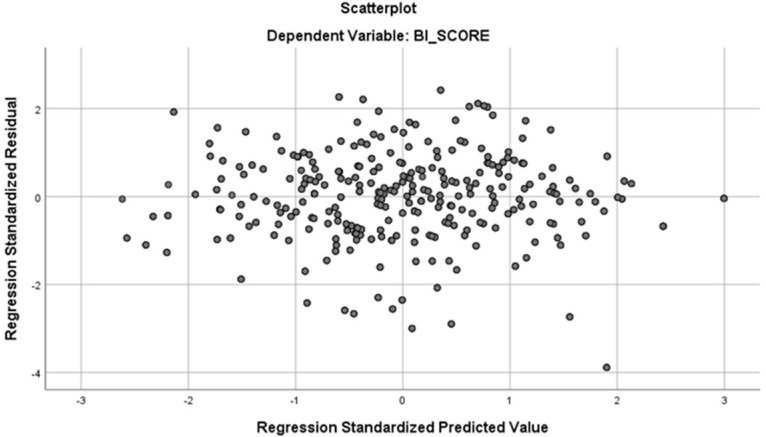
Standardized residuals vs. standardized predicted values.

**Scheme 2:**
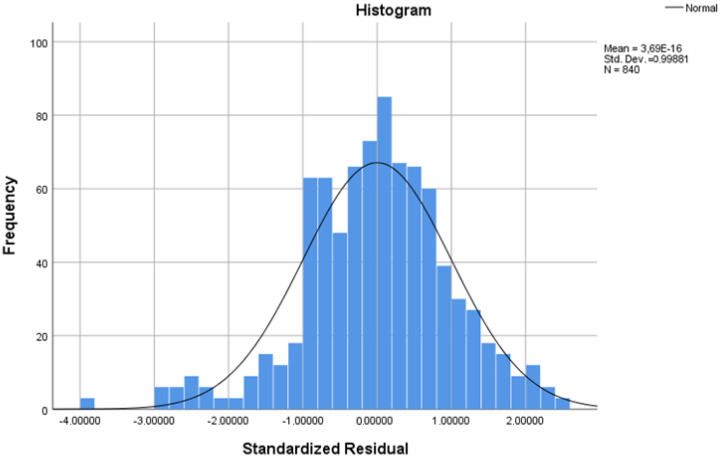
Histogram of unstandardized residuals.

Before running all the regression analyses, regression assumptions were tested: 1.The relationship between independent and dependent variables is linear (valid) [The model correlation table (Table 4) verifies the first assumption]; 2. There is no multicollinearity (valid) [Tolerance (0.856) and VIF (1.169) values (Table 8) are within accepted thresholds. (VIF < 5, Tolerance > 0.2)]; 3. The values of the residuals are independent (valid) [The Durbin–Watson value is 1.991 (Table 8), so this assumption has been met as the obtained value is close to 2]; The variance of the residuals is constant (valid) [In order to test homoscedasticity, a scatterplot was created. The plot (Scheme 1) of standardized residuals vs. standardized predicted values showed no obvious signs of funneling, suggesting the assumption of homoscedasticity has been met.]; The residuals are normally distributed (valid) [Normality cannot be rejected (*p*-value > 0.05) (Table 9). Moreover, we can visually examine that by the histogram (Scheme 2)]; There are no “extreme cases” that influence the model (valid) [The Cook’s distance values should be under 1. In this model, all the Cook’s distance values are within accepted thresholds]. 

**Figure 2:**
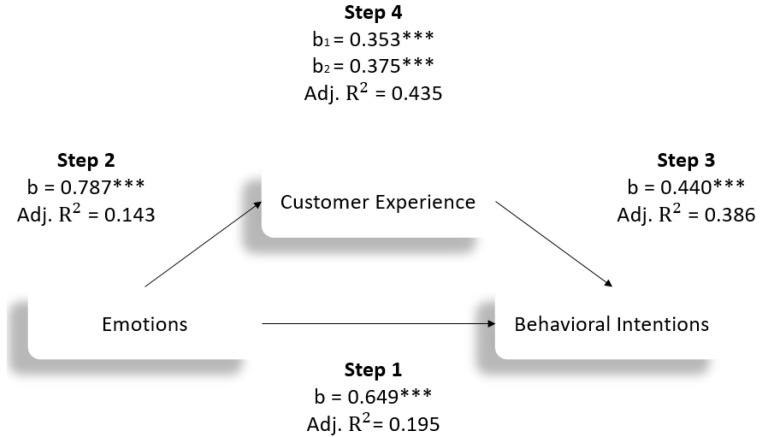
Regression analyses results. *** the significance is lower than 0.001.

As a result, the regression is valid and the mediation is proved, providing support for Research Hypothesis 4 (Figure 2).

The results of the mediation analysis indicated that emotions did affect the behavioral intentions of the customers (Step 1 Adj. R^2^ = 0.195)—with customer experience mediating this relationship—as well as customers’ experience [Step 2 Adj. R^2^ = 0.143). Furthermore, customer experience had a strong impact on customer behavioral intentions (Step 3 Adj. R^2^ = 0.386). Finally, the analysis revealed that the customers’ emotions and experiences greatly affected their behavioral intentions (Step 4 Adj. R^2^ = 0.435) (Figure 2).

As a result of all the analyses, Research Hypothesis 4 was supported, which verified that customer experience does mediate the relationship between customers’ emotions and customers’ behavioral intentions. The model equation is formulated as follows: Behavioral Intentions = 0.340 + 0.353 × Emotions + 0.375 × Customer Experience + e.

## 5. Conclusions and Suggestions for Future Research

The current study had three objectives. The first one was to examine the customer experience and emotion measurement instruments proposed by the scientific bibliography in order to test their applicability in passenger transportation services. The second one was to adapt the behavioral intentions measurement instruments suggested in the literature and create a new measurement instrument and ascertain its applicability in the service under examination. The adapted behavioral intentions scale included items that reflected three behavioral intention elements (word of mouth/recommendation, revisit and repurchase). Finally, the major objective of this study was to examine whether customer experience mediates the relationship between customers’ emotions and behavioral intentions.

According to the results, this study revealed the structures of the customer experience, emotion and behavioral intention constructs regarding the passenger transportation service sector and verified the mediating role of customer experience in the customers’ emotions–behavioral intentions relationship. More extensively, the onboard customers’ experience can be measured through a four-dimensional scale consisting of entertainment, education, escapism and aesthetic factors. Customers’ emotions can be assessed through a two-dimensional scale consisting of positive emotion and negative emotion factors, while the customers’ behavioral intentions can be measured through a one-dimensional scale reflecting their word of mouth, repurchase and revisiting intentions.

### 5.1. Theoretical and Managerial Implications 

Customer experience has been a focal point in management research since it results in customer satisfaction, which is critical for obtaining a competitive advantage [87,88]. Customer experience is a subjective, internal construct over which service providers do not have complete control [2,89], which makes customer experience management a very complex issue [90]. According to academics, understanding the customer experience through time is crucial for businesses [1]; thus, customer experience management is increasingly being considered by businesses as a competitive strategy in the marketplace [91,92]. Customers interact with businesses through a variety of channels and media, and customer experiences are becoming more social. Therefore, to create and deliver good customer experiences, managers must integrate diverse company operations, including external partners [1]. A primary managerial goal is to provide a positive experience to customers, and, as a result of this need, the main aim of practitioners is to be able to manage their customers’ experiences. 

Understanding and assessing customer behavioral intention and its constituent elements (word of mouth/recommendations, repurchase/purchase more and revisit) are also fundamental for managers in many services [9,93] because they allow practitioners to improve their customer experience management [72]. Nowadays, there are thousands of platforms and applications and a myriad of channels through which customers can discuss, communicate and share their experiences [77]. Due to the rise of the internet and social media, face-to-face communications have crossed over into ‘mass-produced’ anonymous conversations [8]. As a result, word of mouth has changed into ‘words of mouths’. Managers who invest in monitoring their customers’ word of mouth can potentially avoid sales decreases or service defamation [94] since studies have revealed that negative word of mouth leads to negative sales outcomes and reputation, while positive leads to positive sales outcomes (repurchase and purchase more) and customer loyalty [8,46,72]. However, the most important argument for managers to invest in evaluation of their customer behaviors is that behavioral intentions are viewed as a forerunner to behavioral loyalty [48].

In this study, it was supported that customer experience mediates the relationship between customers’ emotions and customers’ behavioral intentions. Consequently, from a managerial point of view, we suggest that managers should be willing to improve the behavioral intentions of their customers, not only to improve their customers’ emotional responses but also to ameliorate the entire customer experience. Apart from that, since this research verified the mediating effect of customer experience on the emotions–behavioral intentions relationship, it is crucial for managers who focus on their customers’ loyalty to observe not only the customers’ responses (emotional and behavioral) but also their service attributes (service environment, functionality, employees, etc.). This is because service features affect the customer experience, which impacts customers’ responses (emotional and behavioral) and their resulting loyalty towards the company.

Additionally, our study provides several scholarly contributions. Presently in the literature, no other study examines the structures of the customer experience, emotion and behavioral intention constructs in coastal line shipping services. Thus, we provide academics with a behavioral intention measurement instrument that is adapted to the transport and tourism industry. We have also tested the applicability of the customer experience scale (by adopting Pine and Gilmore’s 1998 experience economy model) and emotions scale (by adopting Kujur and Singh’s 2018 construct) in the same sector and verified their validity. Researchers may benefit from this study since it enables them to utilize the created and tested measurement instruments as foundations for designing and conducting future studies.

### 5.2. Limitations and Suggestions for Future Research 

The present research also has some limitations that can offer recommendations for future research. First, this study did not evaluate whether past customer experiences affected the examined customer experience. As a result, it is crucial for researchers to study this relationship. Second, since the customers’ expectations regarding the provided service were not measured and evaluated in this study, it would be interesting in the future to assess the effects of customers’ expectations regarding a service on the relationships between customer experience and behavioral intentions, between emotions and customer experience and, finally, between emotions and behavioral intentions.

## Figures and Tables

**Table 1:** KMO and Bartlett’s test.

Kaiser–Meyer–Olkin Measure of Sampling Adequacy		0.832
Bartlett’s Test of Sphericity	Approx. Chi-Square	5926.251
	Df	21
	Sig.	0.000

**Table 2:** Total variance explained.

Factor	Initial Eigenvalues			Extraction Sums of Squared Loadings		
	Total	% of Variance	Cumulative %	Total	% of Variance	Cumulative %
1	4.551	65.008	65.008	4.234	60.487	60.487
2	0.932	13.309	78.371			
3	0.616	8.795	87.113			
4	0.394	5.631	92.744			
5	0.371	5.300	98.044			
6	0.118	1.693	99.737			
7	0.018	0.263	100.000			
Extraction Method: Principal Axis Factoring						

**Table 3:** Absolute and incremental fit indices of the examined constructs.

Fit Index	Customer Experience	Emotions	Behavioral Intentions
GFI	0.972	0.982	1.000
AGFI	0.931	0.955	0.998
NFI	0.981	0.976	1.000
NNFI/TLI	0.969	0.963	1.003
CFI	0.985	0.981	1.000
RMSEA	0.067	0.068	0.000
RMR	0.074	0.046	0.002

**Table 4:** Correlations (emotions, customer experience, behavioral intentions).

Model Correlations			
	Emotions	Customer Experience	Behavioral Intentions
Emotions	1	0.380	0.442
Customer experience	0.380	1	0.622
Behavioral intentions	0.442	0.622	1
All correlations are significant at the 0.01 level.			

**Table 5:** Step 1. First simple regression equation of the mediation analysis.

Dependent Variable: Behavioral Intentions			
Adj. R^2^ = 0.195, F-value = 203.882 *p*-value < 0.001	B	t	Sig.
(Constant)	5.516	3.213	0.001
Emotions	0.649	14.279	0.000

**Table 6:** Step 2. Second simple regression equation of the mediation analysis.

Dependent Variable: Customer Experience			
Adj. R^2^ = 0.143, F-value = 141.378 *p*-value < 0.001	B	t	Sig.
(Constant)	13.790	5.514	0.000
Emotions	0.787	11.890	0.000

**Table 7:** Step 3. Third simple regression equation of the mediation analysis.

Dependent Variable: Behavioral Intentions			
Adj. R^2^ = 0.386, F-value = 527.926 *p*-value < 0.001	B	t	Sig.
(Constant)	10.706	12.392	0.000
Customer Experience	0.440	22.977	0.000

**Table 8:** Step 4. The multiple regression equation of the mediation analysis.

Dependent Variable: Behavioral Intentions				
Adj. R^2^ = 0.435, F-value = 323.744 *p*-value < 0.001 Durbin–Watson = 1.991	B	Sig.	Tolerance	VIF
(Constant)	0.340	-	-	-
Emotions	0.353 (b1)	0.000	0.856	1.169
Customer experience	0.375 (b2)	0.000	0.856	1.169

**Table 9:** Normality test unstandardized residuals.

Test of Normality						
	Kolmogorov–Smirnov			Shapiro–Wilk		
	Statistic	Df	Sig.	Statistic	Df	Sig.
Unstandardized Residual	0.052	840	0.000	0.995	840	0.005

## Data Availability

Not applicable.

## Abbreviations

EFA	Exploratory Factor Analysis
CFA	Confirmatory Factor Analysis

## Appendix A. Measurement Instruments

**Customer Experience**
Entertainment
This journey experience was fun
This journey was entertaining
I really enjoyed this travel experience
Education
I learned a lot through my travel experience
This travel experience stimulated my curiosity to learn new things
I completely escaped from reality during this travel experience
Escapism
This travel experience stimulated my curiosity to learn new things
This journey made me feel I was living in a different time or place
I completely escaped from reality during this travel experience
Aesthetic
It was pleasant just being in this ship
The setting of the ship provided pleasure to my senses
The setting of the ship really showed attention to detail in terms of design
**Emotions**
Positive emotions
I feel Contentment (Contended, Peaceful, Fulfilled)
I feel Happy (Optimistic, Pleased, Thrilled, Enthusiastic)
I feel Love (Romantic, Sentimental, Warm-hearted)
Negative emotions
I feel Angry (Frustrated, Irritated, Unfulfilled)
I feel Sad (Depressed, Miserable, Helpless)
I feel Fear (Scared, Afraid, Worried, Nervous)
I feel Ashamed (Embarrassed, Ashamed, Humiliated)
**Behavioral intentions**
I would speak positively about the employees of this shipping company to others
I would recommend traveling with this shipping company to friends
I would recommend traveling with this shipping company to family members
I would mention to others that I traveled with this shipping company
I would make sure that others knew that I traveled with this shipping company
I intend to continue traveling with this shipping company
I plan to travel again with this shipping company in the near future
